# Evaluation of Salivary and Serum Total Antioxidant Capacity and Lipid Peroxidation in Postmenopausal Women

**DOI:** 10.1155/2020/8860467

**Published:** 2020-11-17

**Authors:** Fatemeh Zovari, Hadi Parsian, Ali Bijani, Ameneh Moslemnezhad, Atena Shirzad

**Affiliations:** ^1^Research Committee, Babol University of Medical Sciences, Babol, Iran; ^2^Department of Biochemistry, School of Sciences, Babol University of Medical Sciences, Babol, Iran; ^3^Social Determinants of Health Research Center, Health Research Institute, Babol University of Medical Sciences, Babol, Iran; ^4^Department of Clinical Biochemistry, Facility of Medicine, Babol University of Medical Sciences, Babol, Iran; ^5^Oral Health Research Center, Babol University of Medical Sciences, Babol, Iran

## Abstract

**Objective:**

In menopause, reduction of estrogen hormone affects oxidative stress process in serum. Oxidative stress in saliva plays a significant role in the pathogenesis of oral diseases. The aim of this study was to investigate the total antioxidant capacity and lipid peroxidation in the serum and saliva of premenopausal and postmenopausal women.

**Methods:**

In this case control study, 50 postmenopausal women (case group) and 48 premenopausal women (control group) were selected. The unstimulated whole saliva and serum of the postmenopausal and premenopausal women were collected. The total antioxidant capacity (TAC) of the saliva and serum was measured by ferric reducing antioxidant power (FRAP). Also, malondialdehyde (MDA) was measured by thiobarbituric acid reactive substance (TBARS) method for serum and saliva. Then, the obtained data were analyzed by SPSS 17, whereby Mann–Whitney test and Spearman's correlation test were used. *P* < 0.05 was considered statistically significant.

**Results:**

The postmenopausal group had significantly lower mean serum TAC and higher mean serum MDA than the control group (*P* < 0 < 001 and *P* < 0.01, respectively). The mean salivary TAC and MDA, however, did not differ significantly between the case and control group (*P* = 0.64 and *P* = 0.08, respectively).

**Conclusion:**

In postmenopausal women, with elevation of serum MDA and reduction of serum TAC, the extent of serum oxidative stress grows, but MDA and TAC levels of saliva do not change.

## 1. Introduction

Menopause is a physiological process which usually occurs between 45 and 55 years of age. In this period of a woman's life, permanent pause of follicular activity of the ovary and, eventually, menstruation cycle occurs [1, 2]. During menopause, many women experience different symptoms including hot flashes, cardiovascular disease (CVD), mood disorders, osteoporosis, as well as thinness and dryness of the vagina because of diminished estrogen levels [3, 4]. Oral health also undergoes changes in menopause women, and among the oral complications one can mention xerostomia, burning mouth syndrome, increased incidence of dental caries, dysesthesia, taste changes, gingival atrophy, periodontitis, and jaw bone osteoporosis [5]. On the other hand, some studies have suggested that menopause with imbalance between free radicals and antioxidant activity is considered a risk factor for development of oxidative stress [6, 7]. The reason of oxidative stress in menopause might be diminished levels of estrogen, which is a natural antioxidant by itself, and causes changes in lipid profile and in turn enhanced lipid peroxidation [8]. Studies have indicated that oxidative stress plays a significant role in the pathogenesis of diseases associated with menopause including coronary artery disease, neurological disease, and vasomotor disorders [3, 9].

MDA as the most important product of lipid peroxidation can be used as a marker for the measurement of oxidative stress. Since the body antioxidants are highly varied, using an index that measures total capacity of antioxidants in biological fluids, such as TAC, could be a new and suitable method [10]. The mouth is a critical site for oxidative stress, whose tissue damages usually originate from microbial, chemical, and thermal stimulators. Saliva, as one of the biological fluids of the body and the first line of defense in the oral cavity, fights the activity of oxidants through various antioxidant mechanisms [10].

Additionally, evaluation of saliva is a noninvasive and simple method versus blood in oxidative stress examinations. Furthermore, changes in salivary biomarkers can represent many oral and systemic disorders of the body [11–14].

Since menopause may affect the oxidant-antioxidant system of saliva, and as salivary oxidant-antioxidant imbalance is associated with oral complication due to estrogen changes including xerostomia, dental caries, gingival atrophy, and other conditions, and given the few studies on evaluation of the oxidant-antioxidant system of saliva in postmenopausal women, we examined TAC and MDA in the serum and saliva of postmenopausal women and compared them with premenopausal women.

## 2. Methods

### 2.1. Study Design and Ethics

This case control study was performed on 98 women referring to faculty of dentistry and healthcare centers affiliated to Babol University of Medical Sciences in 2017–2018 (ethics code: MUBABOL.REC.1396.60).

### 2.2. Sample Size and Data Collection

The case group included 50 postmenopausal women within the age range of 41–74 years, while the control group was composed of 48 premenopausal women with the age range of 40–54 years. The inclusion criteria for the case group included the following: postmenopausal women whose menopause had been initiated for at least one year. Menopausal status was self-reported, and duration of menopause is determined from the day that they have been diagnosed. On the other hand, the inclusion criteria for the control group involved premenopausal women with regular menstruation and at least 40 years of age. The exclusion criteria in both groups included intraoral lesions, active dental caries, complete edentulism, severe periodontal disease, dry mouth, pregnancy, cigarette smoking and alcohol consumption, consuming antioxidant supplements (including vitamin and iron supplements), use of topical corticosteroid medications for at least three weeks, use of any kind of hormone replacement therapy, and known systemic diseases (such as diabetes, chronic kidney disease, and cardiovascular disease). Written informed consent form was taken from all women participating in study to collect the samples (unstimulated whole saliva and serum). Once the subjects were selected, the information related to both groups was recorded including age and duration of menopause in the groups.

Two samples were taken from all patients: (1) unstimulated whole saliva and (2) serum. The unstimulated whole saliva sampling was performed through spitting method [15]. In this method, saliva is allowed to accumulate in the floor of the mouth, and the patient spits it out into the graduated test tube every 60 seconds. The samples were collected between 8 and 10 a.m. All patients in the case and control groups avoided eating, drinking, and tooth brushing at least one hour before collection of the saliva. Then, venous blood sampling was performed by a trained nurse, and blood was taken from all patients who had been fasting for 9-10 h. Next, the samples were immediately sent to the biochemistry department of Babol University of Medical Sciences.

### 2.3. Measuring TAC and MDA of Saliva and Serum

In the laboratory, unstimulated whole saliva and serum were centrifuged (Clement 2000, North Sydney, Australia) for 10 min at 2000 rpm. Then, the top filtered solution of the tubes was transferred to coded microtubes, with the samples being kept at −80°C until final biochemical analysis.

The TAC of saliva and serum was measured by the FRAP method [16]. For this purpose, the serum and saliva antioxidants reduced Fe^3+^ in FRAP reagent, whereby color was produced. The working solution of FRAP contained 10 millimolar of TPTZ (2, 4, 6 tripyridyl-s-triazine) in HCl 40 millimolar, FeCl_3_ 20 millimolar, and acetate buffer 0.3 molar (pH = 3.6) at a ratio of 10 : 1 : 1. Then, 1.5 mL of the FRAP working solution was brought to 37°C, and then 50 *μ*L of the sample was added to the solution in order to initiate the reaction. The extent of absorption of the samples and the standard solution of FeSO_4_·7H_2_O containing different concentrations (0–62.5–125–250–500–1000 *μ*molar) was read at the wavelength of 593 nm using a spectrophotometer (UNIC 2100). The antioxidant concentration of saliva was obtained by comparing the optical absorption of the samples with standard solution and in standard curve.

MDA, as a lipid peroxidation product, was measured by TBARS method [17]. For this purpose, MDA reacted with TBA (thiobarbituric acid) at 90–100°C and pH = 2-3 within the reaction time of 15 min, which culminated in a pink pigment. The standard solution (Zellbio Company) was prepared at the concentrations of 0–2.5–5–10–50 millimolar. After preparing the TBARS working solution (containing 1.125 g of TBA and 45 g of TCA) it was brought to the volume of 100 mL by HCl 37%, 500 *μ*L of the samples was mixed with 2 ml of the working solution, and their mixture was placed inside a boiling water bath for 15 min. After the incubation time, the tubes were transferred to a cool water container. Once the tubes cooled down, the samples were recentrifuged at 1500 g for 10 min. Then, the extent of absorption of the samples and standard solution was measured at the wavelength of 535 nm using a spectrophotometer (Jenway, Statfordshire, UK) against the blank. The lipid peroxidation concentration of the samples was obtained through the standard curve.

Note that all chemicals studied here were prepared by Merck Company, Germany.

### 2.4. Statistical Analysis

Then, the obtained data were analyzed by SPSS 17, through Mann–Whitney test and Spearman's correlation test. Mann–Whitney test was used in abnormal distribution. *P* < 0.05 was considered statistically significant.

## 3. Results

The case group had 50 postmenopausal women with the mean age of 56.48 ± 7.62 years, while the control group consisted of 48 premenopausal women with the mean age of 43.65 ± 3.33 years.

In the case group, the mean duration of menopause was 8.34 ± 6.50 years. The mean duration of menopause had a significant correlation with serum MDA (*P* < 0.001, *r* = 0.4), but such a correlation was not observed in serum TAC assessment (*P* = 0.07). Furthermore, in the case group, the duration of menopause had a significant relationship with salivary TAC and MDA (*P* < 0.001 and *P* < 0.001, respectively) (*r* = 0.4 and *r* = −0.4, respectively).

The mean serum TAC in the case group was significantly lower than the mean serum TAC in the control group (*P* < 0.001) (Table 1).

The mean serum MDA in the case group was significantly higher compared with the mean serum MDA in the control group (*P* = 0.01) (Table 1).

The mean saliva TAC and MDA in the case group, however, was not significantly different compared with the mean saliva TAC and MDA in the control group (*P* = 0.64 and *P* = 0.08, respectively) (Table 1).

An inverse correlation between serum TAC and serum MDA, where with elevation of the serum TAC, serum MDA diminished (*P* = 0.04, *r* = −0.28). There was no significant correlation between salivary TAC and MDA levels (*P* = 0.09).

There was no significant correlation between serum TAC and salivary TAC (*P* = 0.27). This was also the case for serum MDA and saliva MDA (*P* = 0.33).

## 4. Discussion

In this study, the serum MDA was higher in postmenopausal women when compared to their premenopausal counterparts. Sa'nchez Rodroguez and Cakir also obtained similar results [18]. MDA, which is the final product of lipid peroxidation, is used as an important characteristic lipid peroxidation both in in vivo and in vitro studies for investigating different types of disease [18]. In this regard, reduction of estrogen levels in menopause can cause oxidative stress [19]. Since during menopause, the follicular activity of the ovary ends, estrogen levels fall, causing changes in the lipid profile, and in turn increased lipid peroxidation [8, 20].

In the present study, the total antioxidant capacity of serum was considerably lower in postmenopausal women when compared to the premenopausal women. Some studies have examined the status of enzymatic and nonenzymatic antioxidants before and after menopause. They reported that, in postmenopausal women, enzymatic antioxidants such as superoxide dismutase, glutathione peroxidase, catalase, as well as nonenzymatic antioxidants such as vitamin C, *α*-tocopherol, and retinol decline, suggest oxidative stress in cells [19, 21]. Although research has measured specific antioxidants and, in the present study, the total antioxidant capacity has been examined, the results of those studies are still in line with the present study. Since, in the antioxidant system of the body, there are various enzymatic and nonenzymatic antioxidants, it seems that all types of antioxidant are impaired in the menopausal process. As it is not possible to identify and investigate every single antioxidant of the body and since antioxidants have a cumulative effect and synergistic reactions with each other, presence of a total antioxidant potential may offer better biological information when compared to investigating every single antioxidant [22]. TAC is the serum total antioxidant capacity, capturing the cumulative and synergistic effect of all antioxidants, which can be a better factor for investigating the antioxidant system in the body.

In the present study, a specific inverse significant relationship was observed between serum TAC and MDA in postmenopausal women. This suggests that, with the increase in lipid peroxidation levels, reduction of total antioxidant capacity occurs. This finding can suggest that, in the oxidative stress process, there is no balance between production of oxidants and the activity level of antioxidants in postmenopausal women, resulting in elevation of ROS levels in biological fluids. The main source of free radicals in the body is physiological metabolism; however, ROS and RNS can also be generated through exposure to external factors such as oral bacteria, ionizing and ultraviolet radiation, food, and air pollution, alcohol, and cigarette smoking. It has been shown that excessive production of ROS in the oral cavity may cause oxidative stress and oxidative damage to cellular DNA, lipids, and proteins, thus predisposing to many oral and systemic diseases [22].

It can also contribute to developing oxidative stress for cells and can play a key role in damaging the function as well as order and integrity of cellular structures. In this way, oxidative stress can play a significant role in the pathogenesis of different diseases associated with menopause including cardiovascular disease, osteoporosis, and hot flashes [3, 4].

This research has been the first study to examine the TAC and MDA levels in the saliva of postmenopausal women. Determining the levels of oxidative stress markers requires use of invasive techniques such as blood sampling [23]. However, in this study, in addition to blood, saliva was also used as a noninvasive and simple means for investigating oxidative stress. In the present study, although saliva TAC levels were lower in postmenopausal women when compared to their premenopausal women, while MDA was greater in the former group, these differences did not prove to be significant. Since, in this study, only the total antioxidant capacity has been examined, while the saliva antioxidant systems are diverse, some special enzymatic or nonenzymatic antioxidants may rise or fall individually, whose cumulative effect does not develop a difference in TAC. Additionally, MDA alone may also have this similar effect, as only lipid peroxidation has been examined, while the oxidation products resulting from DNA molecules and protein may play a more significant role in saliva oxidative stress.

In the present study, there was no significant relationship between serum TAC and saliva TAC. Similarly, there was no significant relationship between serum MDA and saliva MDA either. Notably, no study has examined this relationship so far. Such discrepancies between the results on serum and saliva can be attributed to the following reasons: some types of antioxidants as well as saliva and plasma oxidants are different in terms of chemical composition both quantitatively and qualitatively. In addition, saliva as the first defense line causes lipid peroxidation following chewing and digestion of food. Accordingly, the antioxidant as well as serum and saliva oxidant status is different [24]. Furthermore, the type of disorder and disease may yield different effects on different types of serum and saliva oxidative stress factors. Some studies have reported a relationship between antioxidants and oxidants of serum and saliva in type I and II diabetes mellitus [25, 26]. However, other studies have not found any relationship between antioxidants as well as serum and saliva oxidants in association with cardiovascular disease and temporomandibular joint disorders [27, 28].

The oxidative stress process is considered as an etiological factor for various diseases including CVD, osteoporosis, and ovarian dystrophy, all of which are menopausal symptoms [29]. Currently, it has been established that estrogen is a potential antioxidant, which causes diminished low-density lipoprotein oxidation. The antioxidant mechanism of estrogen is based on its hydroxy phenolic structure, causing removal of free radicals. Additionally, estradiol can cause stimulation of cellular antioxidant enzymes [30]. On the other hand, the effects of oxidative stress in the oral cavity have proven to contribute to chronic inflammatory periodontal diseases, RAS, oral lichen planus, etc. [31–33]. The relationship between serum oxidative stress and menopausal symptoms has been investigated and confirmed. Nevertheless, since no study has been conducted about saliva oxidative stress and oral symptoms in association with menopause, such as periodontitis, dry mouth, and burning mouth syndrome, it is suggested that further studies be conducted in this regard.

## 5. Conclusion

The results of this research suggested that, in menopausal women, with elevation of serum MDA and reduction of serum TAC, the extent of serum oxidative stress rises, but MDA and TAC levels of saliva do not change.

## Figures and Tables

**Table 1:** TAC^1^ and MDA^2^ of saliva and serum of the postmenopausal and premenopausal women.

	Premenopausal women (control), *n* : 48 Mean ± standard deviation Median (IQR)^3^	Postmenopausal women (case), *n* : 50 Mean ± standard deviation Median (IQR)	*P* value
TAC serum (*μ*M)	846.04 ± 152.56	643.31 ± 280.61	<0.001^*∗*^	
	878.3 (809.5–927.5)	752.7 (330.8–883.1)		
MDA serum (*μ*M)	11.98 ± 8.91	12.97 ± 4.24	0.01^*∗*^	
	10.41 (8.67–13.05)	12.58 (10.45–14.52)		
TAC saliva (*μ*M)	474.55 ± 185.66	460.63 ± 222.87	0.64	Mann–Whitney test was used	
	433.6 (352–566.2)	453.6 (281.6–679.5)		
MDA saliva (*μ*M)	6.13 ± 6.30	3.81 ± 2.05	0.08	
	3.72 (2.61–7.14)	3.2 (2.25–4.57)		

^1^Total antioxidant capacity. ^2^Malondialdehyde. ^3^Interquartile range. ^*∗*^*P* value is significant.

## Data Availability

The data (the amount of antioxidants and lipid peroxidation in each patient) used to support the findings of this study are available from the corresponding author upon request.
